# Supplementary material for: COVID-19 Vaccine Hesitancy among Parents of Children with Chronic Liver Diseases
Source: Vaccines (Basel). 2022 Dec 7;10(12):2094. doi: 10.3390/vaccines10122094 (PMC9783335; doi:10.3390/vaccines10122094)
Supplement: Supplementary file 1 [file vaccines-10-02094-s001.zip › Supplementary S2, S3 (additional table and figures).pdf]

Supplementary S2

Supplementary Table S1. Multi-collinearity test for exogenous variables with all endogenous variables

Exogenous Variable Name	Mediator Variable Name				Dependent Variable Name	
	Safety & efficacy		Attitude & Trust		Hesitancy of Parents About Childhood Vaccines	
	Tolerance	VIF	Tolerance	VIF	Tolerance	VIF
Child age	0.948	1.055	0.936	1.068	0.936	1.068
Illiterate	0.774	1.292	0.767	1.304	0.767	1.304
Low educational level	0.830	1.205	0.829	1.206	0.829	1.206
Family size	0.938	1.066	0.927	1.079	0.927	1.079
Income	0.858	1.166	0.852	1.174	0.852	1.174
Child sex	0.968	1.033	0.967	1.035	0.967	1.035

A multi-collinearity test was done for all exogenous variables with all endogenous variables (dependent variable and mediator variables) and no inter-correlation was found where all tolerance values were more than 0.2, also VIF (Variance Inflation Factor) were less than 5, as it is clear in the following table supplementary table 1. The following two figures supplementary figure 1 and 2 emphased that there is no heteroscdecity, on other words the variance around the regression line is the same for all values of the predictor variable so the assumption of homoscedasticity is met.

Supplementary S3

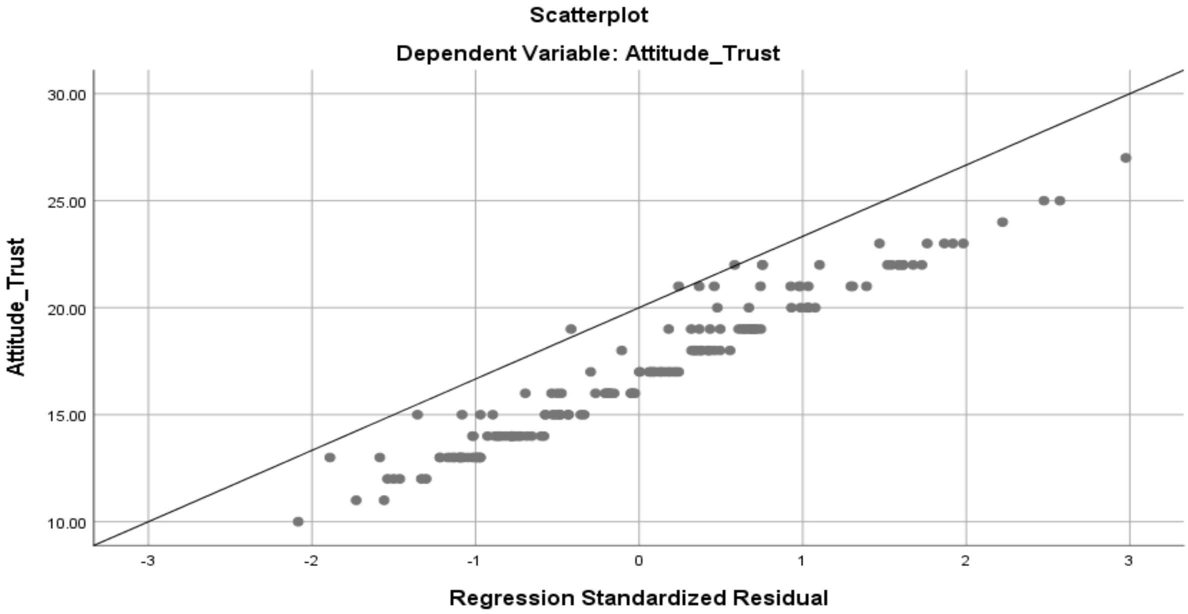

Supplementary Figure S1. Scatter Plot for the relation between the regression standardized residual and the dependent variable Attitude and Trust

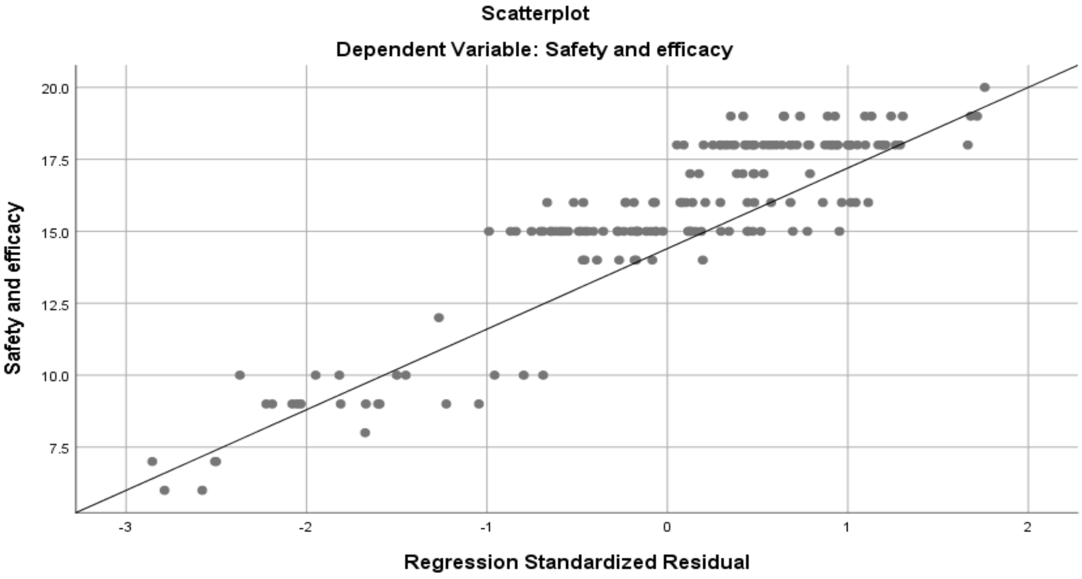

Supplementary Figure S2. Scatter Plot for the relation between the regression standardized residual and the dependent variable Safety and Efficacy
